# Changes in field workability and drought risk from projected climate change drive spatially variable risks in Illinois cropping systems

**DOI:** 10.1371/journal.pone.0172301

**Published:** 2017-02-23

**Authors:** Bradley J. Tomasek, Martin M. Williams, Adam S. Davis

**Affiliations:** 1 Department of Crop Sciences, University of Illinois at Urbana-Champaign, Urbana, Illinois, United States of America; 2 United States Department of Agriculture/Agricultural Research Service, Global Change and Photosynthesis Research Unit, Urbana, Illinois, United States of America; Instituto Agricultura Sostenible, SPAIN

## Abstract

As weather patterns become more volatile and extreme, risks introduced by weather variability will become more critical to agricultural production. The availability of days suitable for field work is driven by soil temperature and moisture, both of which may be altered by climate change. We projected changes in Illinois season length, spring field workability, and summer drought risk under three different emissions scenarios (B1, A1B, and A2) down to the crop district scale. Across all scenarios, thermal time units increased in parallel with a longer frost-free season. An increase in late March and Early April field workability was consistent across scenarios, but a decline in overall April through May workable days was observed for many cases. In addition, summer drought metrics were projected to increase for most scenarios. These results highlight how the spatial and temporal variability in climate change may present unique challenges to mitigation and adaptation efforts.

## Introduction

Agriculture faces considerable challenges over the next century. In addition to the already competing challenges of feeding a growing global population and increasing environmental sustainability [1], global climate change is expected to further complicate future agricultural production and management decisions. Net socio-economic effect of climate change on agriculture will differ from region to region including both positive and negative net effects [2]. For example, increases in temperature may allow new cultivars that utilize longer frost-free seasons and a greater number of growing degree days (GDDs) for greater yields. On the other hand, increases in temperature increase evapotranspirative demand. If this higher atmospheric demand for water is not met by additional precipitation or irrigation, there is a potential for increases in water stress and drought. Globally, climate change is projected to have a negative impact on global food security [3].

Four types of agricultural adaptation strategies to climate change have been identified: technological advances, government policy and insurance, farm production practices, and farm financial management [4]. Technological innovation has historically played an important role in reducing some of the risks of weather variability to agriculture [5]. However, weather variability is still a key factor driving risks in agriculture. These risks are not limited to the effect of average weather conditions on plant growth. They also include the catastrophic effects of extreme weather, changes in field workability, and the more indirect effects on yield through the responses of pests and weeds [6,7]. Since technological advances alone have not offset the risks associated with weather variability, adaptations to climate change will require a more integrated approach. An integrated approach needs to include both adaptation by the production system and socio-economic adaptations in market risk taking and policy [8]. Considerable effort is now being spent on assessing these potential risks through modeling efforts [9,10].

Field operations in crop production follow a strict timeline based on the relations among soil conditions, crop developmental stages and local weather patterns. The available days suitable for agricultural operations each growing season, or field working days (FWDs), is an example of how weather variability affects risks in agriculture. These risks have economic significance and are often mitigated through a combination of management timing and machinery size selection [11–13]. The availability of FWDs is primarily driven by soil moisture, where soil moisture over a certain threshold is deemed too wet to work [14]. Different soils are assumed to be have the same threshold for workability if the volumetric soil moisture is measured as a percentage of either the field capacity (FC) or plastic limit (PL) of the soil [7]. For example, there is theoretical justification for an optimal soil moisture threshold for tillage of 90% of the PL of a soil [15].

Studies looking at the implications of climate change on the U.S. Midwest from the agricultural perspective draw several consistent conclusions. Growing season length is projected to increase in both accumulation of thermal time units (GDDs) and time between last and first frost dates [16–18]. Winter and spring precipitation is consistently projected to increase in coming decades, resulting in greater soil moisture in the early season, while increased evapotranspiration will decrease soil moisture during the summer [19–26]. Wetter springs will likely make timely spring planting operations difficult and drier summers will increase drought risk [22,27]. Few recent studies explicitly model changes in field workability resulting from climate change [28–31], despite the clear connection between climate and field workability [32].

There were two primary objectives of this study: (1) to determine the effects of projected climate change scenarios on field workability in the state of Illinois and (2) discuss how these changes may interact with projected changes in season length and water availability to influence risks caused by weather variability. Based on previous projections from climate models, we expected to see decreases in field workability due to wetter winter and springs, and greater prevalence of drought risk. We also expected temperature-driven increases in season length defined by GDDs and frost-free season. These changes were examined at two spatial scales. We use the finer resolution of the crop district scale to highlight the spatial variability associated with climate change impacts. We also present results at the broader state average scale, so that overall trends can be summarized.

## Materials and methods

### Field workability data

**Fig 1:**
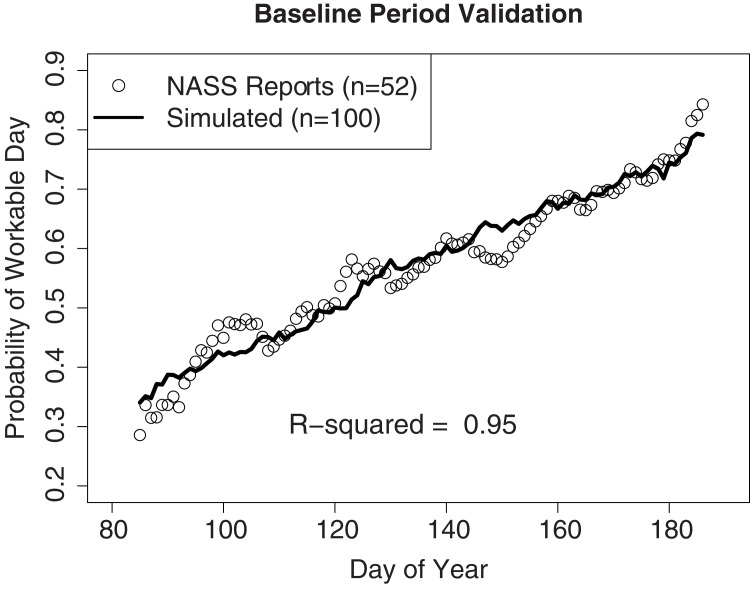
Goodness of fit of the simulated statewide average probability of a field working day. Predictions of the probability of a field working day during the 1960–2000 baseline period (black line) from LARS-WG versus the probability determined by USDA-NASS weekly crop progress reports from 1959–2010 (open circles).

Historical data on the weekly number of FWDs for Illinois were reported by the USDA National Agricultural Statistics Service at both the statewide average and crop district scale [33]. The reports do not specify which days were workable, but the aggregate number of FWDs that were available in seven day periods, with reports providing information from early April through June. The reports contain information at the state average scale from 1959 to 2010, and crop district scale starting in 1980. Statewide FWDs are calculated as the average of the nine crop districts. The statewide average workability probability in Fig 1 was naively calculated by assigning the weekly number of FWDs reported to each day in that period uniformly.

### Daily weather data

Daily weather time series containing precipitation, minimum, and maximum temperature provided by the Cooperative Observer Network were retrieved from the National Climatic Data Center [34]. A total of 50 weather stations from across the state of Illinois were selected based on data availability coinciding with the field workability observations between 1959 and 2010. The number of stations contained within each crop district ranged from three in the East district to eight in the East Southeast district.

Weather time series were also simulated using the weather generator LARS-WG [35]. For the analysis used to train LARS-WG, one station was selected from each crop district as a representative of the conditions for that district. The LARS-WG training period selected extended from 1960 to 2000 with the exception of the Northeast and Central districts starting in 1967 and 1978 respectively. Each station was the one with the lowest average pairwise squared difference in daily precipitation values from all other stations in that district.

### Soil information

Soil texture and organic matter content measurements were obtained from the USDA National Conservation Service Soil Characterization Database [36]. These parameters were necessary to run the soil moisture model and estimate the PL of each soil using equations from [37].

### LARS-WG

Stochastic weather generators are models capable of analyzing historical weather time series and simulating new and independent weather time series which is statistically similar to the training (baseline) period [38]. LARS-WG (version 5.0) was the stochastic weather generator used in this study because it is well suited and easy to implement for agricultural risk assessments [39]. By default, LARS-WG can only generate single-site time series of daily weather. LARS-WG contains a statistical downscaling component to allow for site-specific climate change projections based on one of 14 global circulation models (GCM) used in the IPCC AR4 assessment [40]. While a multi-model ensemble approach would be ideal, we chose the Community Climate Model Version 3 (CCSM3) due to computation time. CCSM3 was specifically chosen because it was (1) among the highest resolution, (2) among newest available GCMs in LARS-WG, and (3) was in the middle range of climate sensitivities to atmospheric carbon [40,41]. The procedures of LARS-WG are explained in the user manual [35].

CCSM3 projections for two different time periods (2046–2065 and 2080–2099) and three different IPCC emissions scenarios B1, A1B, and A2 were used [42]. The differences among scenarios (already built into LARS-WG) are complex and involve socio-economic as well as energy-use considerations, but generally the intensity of climate change is expected to be lowest in the B1 scenario, intermediate in A1B, and highest in A2.

For each region, scenario, and time period (including the baseline period), 1000 years of simulated weather was generated. Due to computation time, only 100 years per region, time horizon, and scenario were randomly selected and run through the soil moisture model to be used in FWD predictions.

### Soil moisture estimation

A batch-run version of the Soil Temperature and Moisture Model (STM^2^), was used to estimate the 10 cm-depth daily soil moisture throughout the study [43]. STM^2^ has low input requirements which include; daily minimum and maximum temperatures, precipitation, soil texture, organic matter content and location.

Daily soil moisture conditions across the state were reconstructed over the historical period of 1959–2010. First, the National Cooperative Soil Survey [36] was used to identify 1–3 soils representative (and with the necessary texture data reported) of agricultural fields within a few kilometers of each weather station. The daily weather time series were run through STM^2^ with each of their associated soils (n = 97) to reconstruct daily soil moisture conditions at each location.

In the case of the LARS-WG output for the climate change scenarios, there is only one station per each crop district (a total of nine). The weather stations not included are assumed to have the same weather as the representative station from their corresponding district. This is a simplification, which underestimates the spatial variability within a crop district, but allows for all the soils to be used.

### Optimized field workability model

The model follows the methods outlined in previous work for predicting FWDs but summarized below [44]. A day was predicted workable only if scaled soil moisture was below a certain threshold (T_s_) and the average temperature was above a different threshold (T_t_). Mathematically this can be represented by Eq (1) where *I* is the indicator function taking value 1 if the statement is true, and 0 otherwise.

$$Work\;Day=I\left(SoilMoisture\leq T_{s}\;and\;Temp\geq T_{t}\right)$$ [1]

The number of FWDs predicted within each district was computed as the average number of FWDs predicted at each weather station in that district. Weather station scale FWDs were predicted as the average number of FWDs predicted for each of the soils associated with that station. An optimization procedure was used to identify the field workability threshold in terms of PL using the reported workable days. Different soil moisture and temperature thresholds were tested iteratively to minimize the squared prediction error of the USDA-NASS reported FWDs.

The procedure was improved to eliminate trends in the prediction error over day of year. Within-season trends in errors were eliminated by splitting the training data into two time periods to create a trending threshold for workability. Period one included weekly reports issued before day of year (DOY) 105, and period two included reports between DOY 105 and 150. The resulting optimal soil moisture and temperature thresholds for the two periods were connected with a simple trend line fit from DOY 105 to 150 and extrapolated past these points (S1 and S2 Figs). The fitted threshold for soil moisture was found to increase through the season and was between 0.7 and 1 times the soil-specific PL (S1 Fig). Temperature thresholds were more variable, but showed similarly increasing trends (S2 Fig). Extrapolation of temperatures thresholds were constrained to be within 0°C and 10°C (S2 Fig).

### Data management and analysis

Data management and analysis was performed in R [45]. The SPEI package [46] was used for calculations of monthly potential evapotranspiration (PET) based on the Thornthwaite equation (S1 Equation) [47]. The Thornthwaite equation is among the simplest models for PET as it only requires temperature and latitude and is based on empirical, rather than mechanistic, model fitting [48]. A more advanced methodology for calculating PET, such as the Penman-Monteith equation, would be ideal. However, such calculations would require inputs (eg. cloudiness, wind speed, and humidity) not available as outputs of LARS-WG. Using the Thornthwaite equation allowed for the calculation of PET with only the output available from LARS-WG. In addition, there is evidence that at monthly and yearly timescales, the Thornthwaite equation performs similarly to the Penman-Monteith equation in both qualitative and quantitative measures of drought in this region [49]. For these reasons, this simple equation is still used with success as a component in some climatological drought metrics, especially when data is limited [50,51].

**Fig 2:**
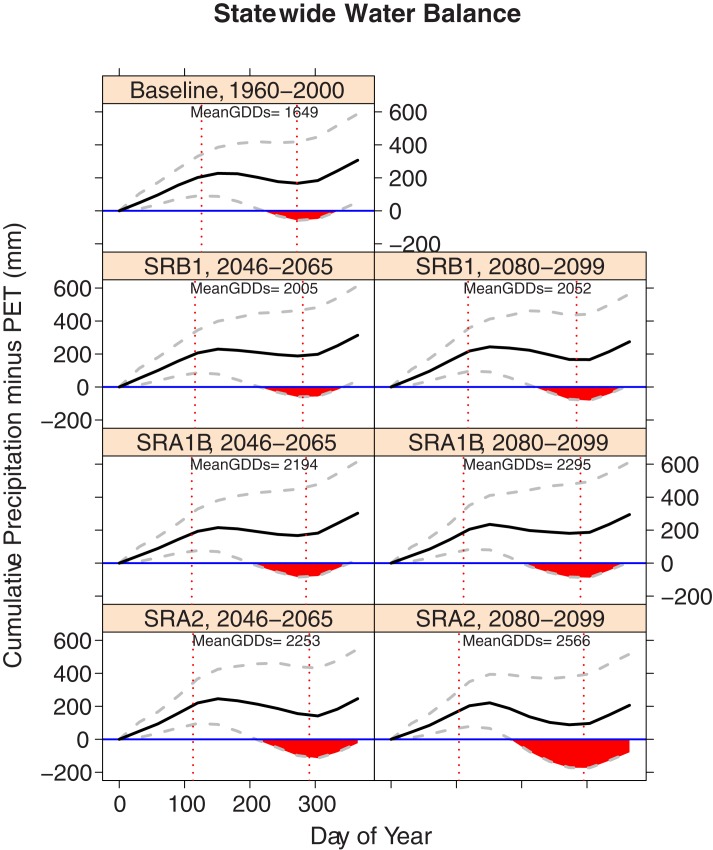
Simulated cumulative average water balance of Illinois. Potential evapotranspiration is calculated using the Thornthwaite equation. All values are calculated as the mean of the individual district mean values with water balance calculated at the end of each month with a linear interpolation between months. The black line indicates the average monthly cumulative water balance. Grey lines indicate the average 95^th^ and 5^th^ quantiles of monthly cumulative water balance with area in which the lower quantile goes below zero marked with red polygons. The dotted vertical lines identify the 90^th^ percentile fall and spring frost dates. Mean GDDs were calculated in degrees Celsius with maximum daily temperature of 30°C and minimum daily temperature of 10°C.

**Fig 3:**
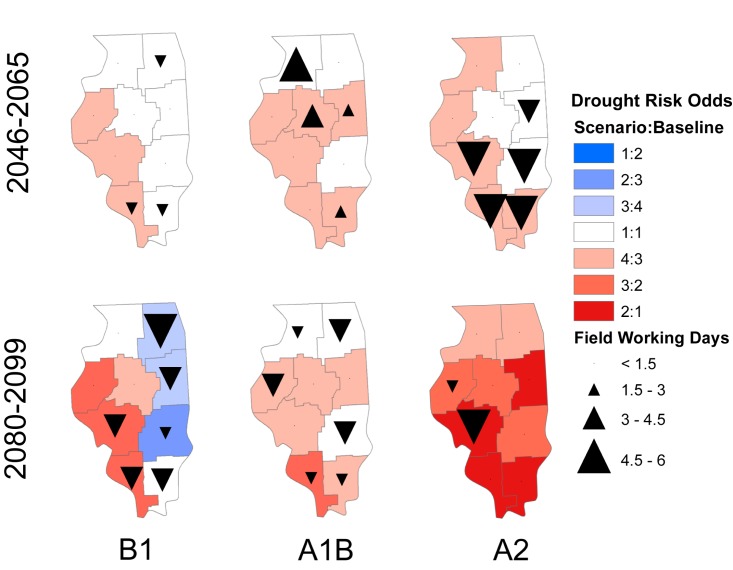
Changes in the number of months in deficit between May and August to the baseline simulation period of 1960–2000 for three different climate change scenarios. Projections are driven by NCAR CCSM3 and downscaled using LARS-WG. Each district contains 1000 years of simulation for each scenario and time horizon. Field working day changes are relative changes in mean number of April through May field working days from the simulated baseline. Arrows pointing up indicate an increase in average number of field working days while those pointing down indicate a decrease.

The drought measure used in this study is the monthly precipitation minus the PET (hereafter called the water balance). This monthly measure is drawn as an accumulation initialized at zero over each year in Fig 2. Months where the cumulative water balance goes below zero are considered to be in deficit. For the drought risk in Fig 3, only the months of May through August are considered and the risk is calculated as Eq (2) with n = 1,000 for every region, scenario, and time period. All results are comparisons relative to the simulations from baseline weather conditions. In this way, we make effort in controlling the effects of modeling assumptions.

$$Risk\;Odds=\frac{{\left(\#\;Months\;in\;Deficit\right)}_{Scenario}}{{\left(\#\;Months\;in\;Deficit\right)}_{Baseline}}$$ [2]

## Results

The LARS-WG diagnostics indicated that nearly all of the simulated weather characteristics had similar distributions to the training data. A few significant differences were detected between training period and simulated weather characteristics in the simulated heat waves, defined as runs of days with temperatures over 30°C, particularly in spring. However, these p-values could change in significance based on the random seed of the generated weather. This indicates discrepancies in the distribution of simulated and observed heat waves may be partly caused by randomness, since these events are rare in the data. The output from LARS-WG was further validated by comparing historical FWD probabilities from USDA-NASS reports to those simulated in the baseline period by LARS-WG. LARS-WG and the optimized field workability model perform exceptionally well (R^2^ = 0.95) at reproducing the historical statewide average field workability probability (Fig 1). Validating on the crop district level, the procedure performed well in five of the nine districts and but has some biases in the remaining four (S3 Fig). One explanation is that the spatial variability of weather patterns in these districts may be poorly represented by the representative weather site. While these four districts do contain some biases, the biases are relatively small (<10% per day on average), vary in sign, and occur at different points in the season which indicate that any errors are not systematic issues introduced by the modeling approach.

**Fig 4:**
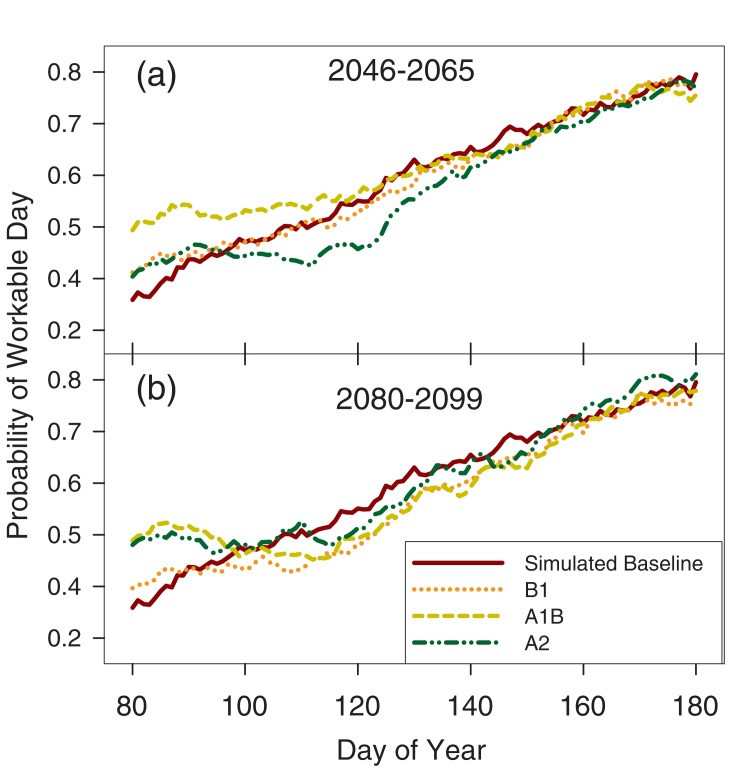
Simulated state average probability of a field working day over day of year. Projections are for four different climate scenarios (Baseline, B1, A1B, A2) and under two different time horizons: (a) years 2046–2065, and (b) years 2080–2099.

The ability of the simulated weather to reproduce the historical statewide probability of FWDs confers some confidence in the validity of the projections. Even so, projections for field workability (Fig 4) and water balance (Fig 2) should be taken in context of relative change from the simulated baseline. Since the modeling approach is the same for the baseline and projected emissions scenario simulations, relative comparisons control any systematic uncertainties or biases that may be present as a result of modeling assumptions.

### Mid-Century (2040–2065)

The combination of earlier last spring frosts (minimum daily temperatures < 0°C) and later first fall frosts are projected to add 20–30 additional days to the length of the growing season (Fig 2). A combination of this longer season and rising daily temperatures correspond to an increase in GDDs. Averaged over the state, these increases range from an additional 350 GDDs Celsius under B1 to an additional 600 GDDs under A2. These projections are similar to previous results for the U.S. Midwest even when different GCMs were used in the downscaling process [17,18].

Under the B1 emissions scenario, state average field workability is expected to experience a small increase in late March workability but otherwise remain similar to historical levels (Fig 2). Small decreases in the average number of April through May FWDs are expected for the southern most districts and the Northeast district (Fig 3). Under the A1B scenario, state average field workability from May through June remains similar to historic probabilities but considerably higher probabilities are forecasted in late March and April (Fig 4). This increase is especially prevalent in the Northwest district with an additional 6 April-May FWDs projected on average. Conversely, the simulated probability of workable days under the A2 scenario is projected to be about 10% lower between the last two weeks of April and first two weeks of May. This decrease owes to the sharp decrease in average April and May FWDs forecasted for the districts in the southern half of Illinois (Fig 3).

Modest increases in the state average probability and severity of a water balance deficit in late summer are observed, with the smallest increases projected for B1 and the greatest for A2 scenario (Fig 2). At the district level the pattern is similar, except under B1 where districts on the eastern half of the state appear to be transitioning to the smaller deficit risk projected by end of the century (S1 File). Among all the scenarios the West, West Southwest, and Southwest districts consistently show increased odds of deficit risk (approximately 4:3) compared to the baseline.

### End of century (2080–2099)

By the end of century, different climate scenarios diverge considerably. Under the less intense scenarios of B1 and A1B, only 50–100 GDDs are added to the state average from the Mid-Century (Fig 2). Under A2, the earlier last spring frost is responsible for most of the 1–2 week increase in the length of the season, corresponding to a 300 GDD increase from the mid-century value. Compared to the baseline period, these changes represent a 3–8 week increase in season length and 500–900 additional GDDs depending on the scenario.

State average field workability projections show increases under A1B and A2 for late March and early April compared to baseline (Fig 4). Across the state and under all scenarios, the number of FWDs available in April and May either remains similar to the baseline period or decreases (Fig 3). For B1 and A1B, decreases in the number of April to May FWDs are expected across much of the state. Under A2, the average number of April to May FWDs shows a sharp decrease in the West Southwest district and a small decrease in the West district, but little change elsewhere.

The state average water balance shows small increases in deficits for B1 and A1B compared to mid-century. There is some spatial variability under the B1 scenario with the southwestern region of the state expected to experience an increase in drought risk and the eastern half of the state expecting a decrease in risk (Fig 3). The projection for the A2 scenario shows large increases in the probability and severity of water balance deficits (Fig 4) with this increased risk spread across every district (Fig 3).

## Discussion

Our simulations consistently predicted an increase in late March and early April field workability. This small increase, coupled with the robust projection of an earlier last frost date, may allow farmers to plant earlier. On the other hand, with the exception of the mid-century A1B scenario, the average number of cumulative April through May FWDs is projected to remain stable or decrease. Projected constraints on mid-spring FWDs are consistent with previous studies projecting higher spring soil moisture caused by increased spring rainfall [18,21,23]. This pattern of decreased field workability is especially pronounced in scenarios for southern Illinois. Less FWDs could make timely field operations more difficult in comparison to the northern part of the state. These changes in field workability may necessitate regional changes in crop choice; variation in field workability has been identified as a driver in the spatial distribution of cropping practices in Europe [52]. Extremely wet conditions during planting have also been shown to negatively affect maize and soybean yields in the U.S. Corn Belt [53]. As a result, cropping practices which allow for more flexible management timing or possess smaller yield potential penalties for delayed spring field operations may become more attractive for the risk-adverse.

Another clear pattern is an increase in probability, severity, and spatial extent of drought risk. Despite the simplicity of the modeling approach and the underlying Thornthwaite PET equation, these results mirror previous research projecting a decrease in summer soil moisture driven largely by increases in evapotranspiration and run-off [18,19,22]. These results also concur with recent efforts using newer climate scenarios and GCMs [24–26]. Specifically, the higher emission scenarios (A1B and A2) show more districts with drought risks, and a higher probability of drought risks from the mid-century to the end of the century. This concurs with the projected temperature increases between the middle and end of the century in all scenarios [40]. However, we note that this increase in drought risk is primarily driven by changes to ET. As a result, some of this projected risk could be alleviated by ET-driven precipitation recycling [54,55]. Irrigation could allow upwind sources to provide increases in precipitation to Illinois in the future that are not accounted for in CCSM3 [56]. Further, even without irrigation this potential source of additional precipitation could be influenced by land-use strategies [57]. This notion of precipitation recycling represents yet another source of uncertainty in our forecasts. As our understanding of this feedback improves, there is a potential for this knowledge to be used to improve long-term weather and field workability forecasts [58].

Previous modeling efforts have shown increases in weather variability are likely to be associated with decreases in maize yields over most of the state, and these changes interacted with GDD day requirements [16,53]. The widening quantile range in the cumulative water balance for the most climate change scenarios in Fig 4 shows such an increase in variability. Considering water availability as a cumulative time series confers another advantage when viewed on a monthly temporal resolution. This perspective highlights risk of increased drought, relative to the baseline scenario, concentrated at the end of the summer. At the state level, this drought risk is evident both earlier in the season, and generally at higher intensity under all of the scenarios.

Given that drought stress can account for a significant proportion of historical average yield losses in U.S. maize, improvements in water-stress tolerance through breeding have an opportunity to play an important role in reducing yield variability [59]. However, even increases in drought tolerance at the individual plant level might not always translate into increased average yields due to increased sowing densities typical of modern field crop production [60]. Understanding how these factors will contribute to regional-scale agricultural productivity also requires knowledge of interactions between changes in field workability and drought risks.

Our analysis indicates that early planting operations in late March and early April may be facilitated by increases in field workability and a retreating spring frost date. From a management perspective, this earlier planting might allow for risk mitigation by allowing critical growth stages (i.e., reproductive stages) to precede the most severe periods of drought risk present in the late summer. Operations dependent on April and May FWDs may be impaired in some districts. This is particularly true under more intense scenarios such as A2 where substantial increases in drought risk are often coupled with decreases in field workability.

We identified three potential responses proposed by these projections that decision-makers might consider as part of a climate-resilient agricultural production strategy. First, plant longer maturity cultivars that match the new season length in terms of GDDs and frost dates. Aided by an increase in late March and early April FWDs, potential yield may be enhanced by cultivars utilizing this longer season. However, this greater yield potential may be difficult to realize under more frequent and intense drought risk. Second, plant early, but instead select a shorter-season cultivar to reduce the risk of encountering late-summer drought conditions at reproductive stages. This response trades maximum possible return (and likely average return) for a decrease in yield variability. Finally, adopt agronomic management practices that conserve soil moisture and select crops with greater drought tolerance. This response would require the greatest changes from contemporary practices and as such is the most extreme mitigation response.

## Conclusions

The key conclusion of this study is that specific impacts of climate change on weather and crop production are likely to be highly variable across Illinois. Specifically related to our study objectives, we have shown that climate change projections suggest changes to (1) the timing and number of workability and (2) the availability of water and drought risk. Even at scales smaller than the state level, significant differences in the magnitude and direction of projected changes were observed. Changes were highly dependent on the greenhouse gas emissions scenario forcing climate change. In general, we projected an increasing trend in the frequency and spatial extent of drought risk through years and across all but the least severe scenario (B1). Models also predict a high level of variability in changes to field workability. Warmer spring temperatures may allow for a few more FWDs in late March and early April under most scenarios. However, the average number of overall FWDs between April and May show large decreases in several crop districts and scenarios. Only one scenario (A1B, mid-century) shows an increase in April to May FWDs. To conclude, changes in weather-related risks caused by global climate change will likely force changes in management practices; either indirectly through the adoption of mitigation strategies, or directly by modifying the availability of workable days.

## Supporting information

S1 Equation The Thornthwaite equation for monthly PET. (DOCX)

S1 Fig Regional daily soil moisture threshold determined by optimization over day of year for field workability. (TIF)

S2 Fig Regional daily mid-range temperature threshold determined by optimization over day of year for field workability. The temperatures thresholds are constrained between 0 and 10° C.
(TIF)

S3 Fig Goodness of fit of the simulated probability of field working day for each crop reporting district. Predictions are made from weather data trained on 1960–2000 from LARS-WG (black line). Observed probabilities are determined by USDA-NASS weekly district crop progress reports from 1980–2010 (open circles).
(TIF)

S1 File Simulated cumulative average water balance of Illinois crop reporting districts as calculated using the Thornthwaite equation for potential evapotranspiration. Water balance is calculated at the end of each month with a linear interpolation between months. The black line indicates the average monthly cumulative water balance. Grey lines indicate the average 95^th^ and 5^th^ quantiles of monthly cumulative water balance with area in which the lower quantile goes below zero marked with red polygons. The dotted vertical lines indicate that 90^th^ percentile fall and spring frost dates. Mean growing degree days calculated in degrees Celsius allowing maximum daily temperature of 30°C and minimum daily temperature of 10°C.
(DOCX)

S1 Table Weather stations extracted from NCDC and used in the analysis. (XLSX)

S2 Table Soil information from US Soil Survey used in the analysis. (XLSX)
