# Supplementary material for: Changes in field workability and drought risk from projected climate change drive spatially variable risks in Illinois cropping systems
Source: PLoS One. 2017 Feb 23;12(2):e0172301. doi: 10.1371/journal.pone.0172301 (PMC5322927; doi:10.1371/journal.pone.0172301)
Supplement: S1 Equation — (DOCX) [file pone.0172301.s001.docx]

The Thornthwaite equation for monthly PET is as follows:

$$PET=16(\frac{L}{12})(\frac{N}{30}){(\frac{10T_{M}}{I})}^{\alpha}$$

Where T_M_ is the average daily temperature for the month

N is the number of days in month M

L is the average day length in hours for month M (determined by latitude)

And parameters α and X are defined as below.

$$\alpha=\left( 6.75 \times{10}^{-7} \right)X^{3}-\left( 7.71 \times{10}^{-5} \right)X^{2}+\left( 1.692 \times{10}^{-2} \right)X+0.49239$$

$$X= \sum_{i=1}^{12} {(\frac{T_{i}}{5})}^{1.514}$$
