# Supplementary figures and images for: Changes in field workability and drought risk from projected climate change drive spatially variable risks in Illinois cropping systems
Source: PLoS One. 2017 Feb 23;12(2):e0172301. doi: 10.1371/journal.pone.0172301 (PMC5322927; doi:10.1371/journal.pone.0172301)

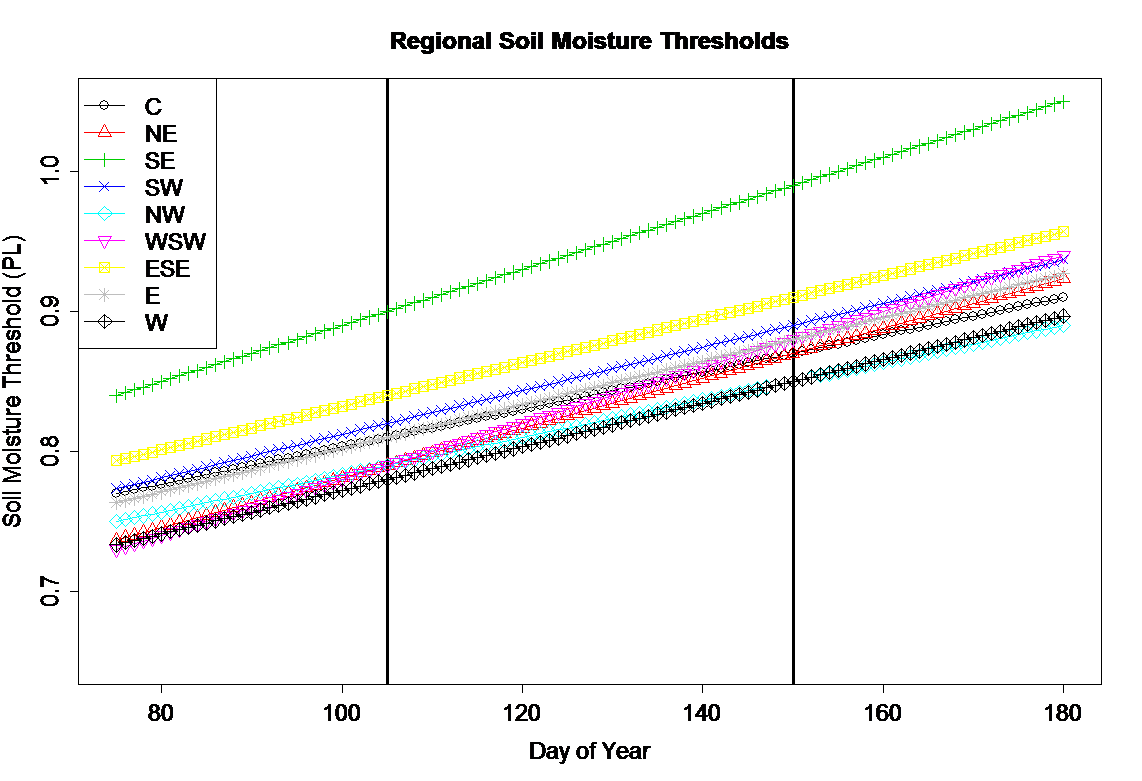

Supplement: S1 Fig — (TIF) [file pone.0172301.s002.tif]

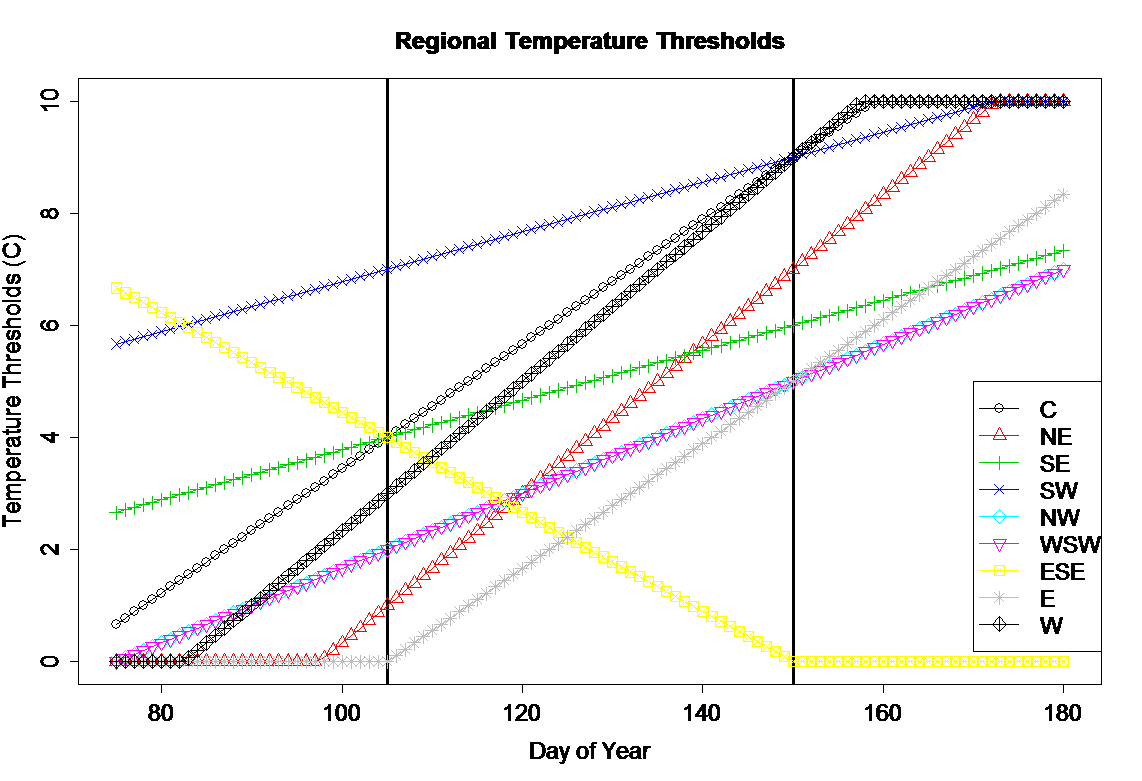

Supplement: S2 Fig — The temperatures thresholds are constrained between 0 and 10° C. (TIF) [file pone.0172301.s003.tif]

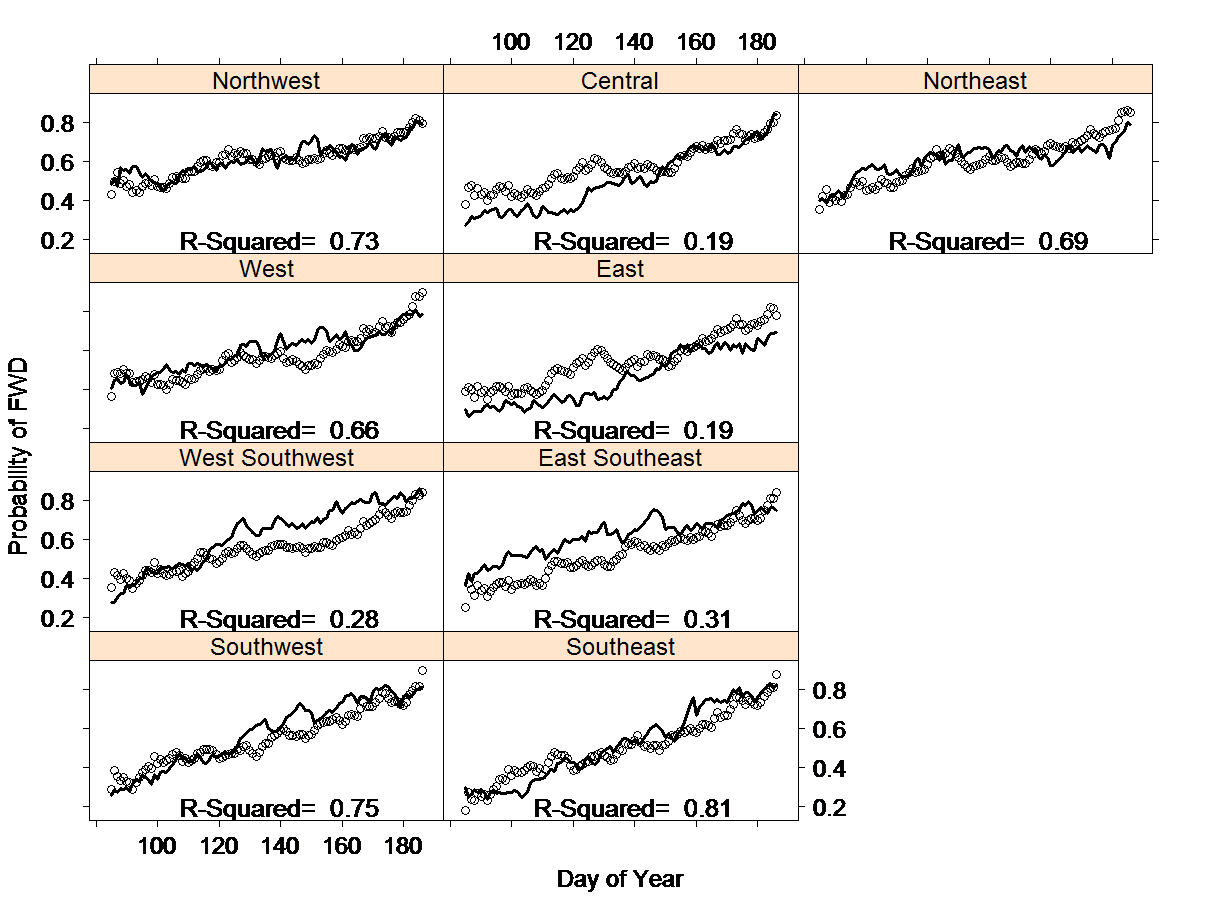

Supplement: S3 Fig — Predictions are made from weather data trained on 1960–2000 from LARS-WG (black line). Observed probabilities are determined by USDA-NASS weekly district crop progress reports from 1980–2010 (open circles). (TIF) [file pone.0172301.s004.tif]
