# Supplementary material for: Changes in field workability and drought risk from projected climate change drive spatially variable risks in Illinois cropping systems
Source: PLoS One. 2017 Feb 23;12(2):e0172301. doi: 10.1371/journal.pone.0172301 (PMC5322927; doi:10.1371/journal.pone.0172301)
Supplement: Supplementary file 3 [file pone.0172301.s005.docx]

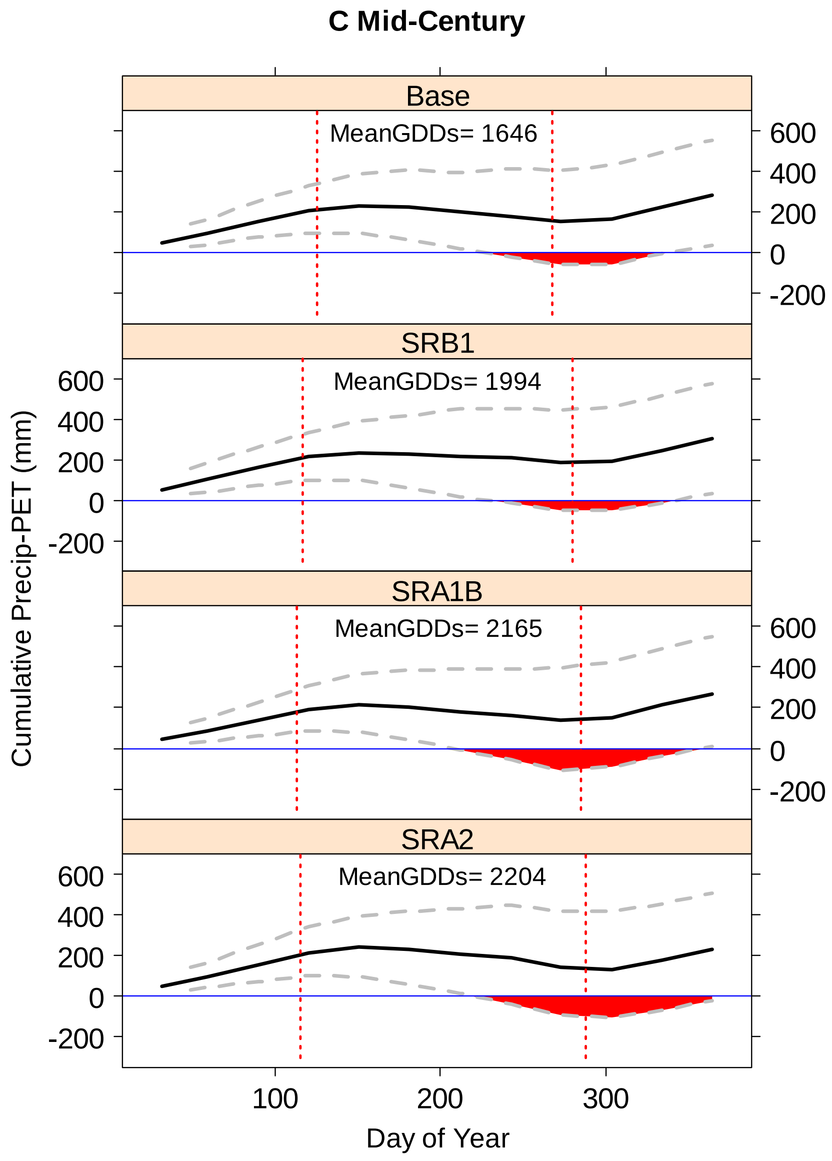
C Mid-Century
Base
MeanGDDs= 1646
SRB1
MeanGDDs= 1994
SRA1B
MeanGDDs= 2165
SRA2
MeanGDDs= 2204
Cumulative Precip-PET (mm)
600
400
200
0
-200
100
200
300
Day of Year

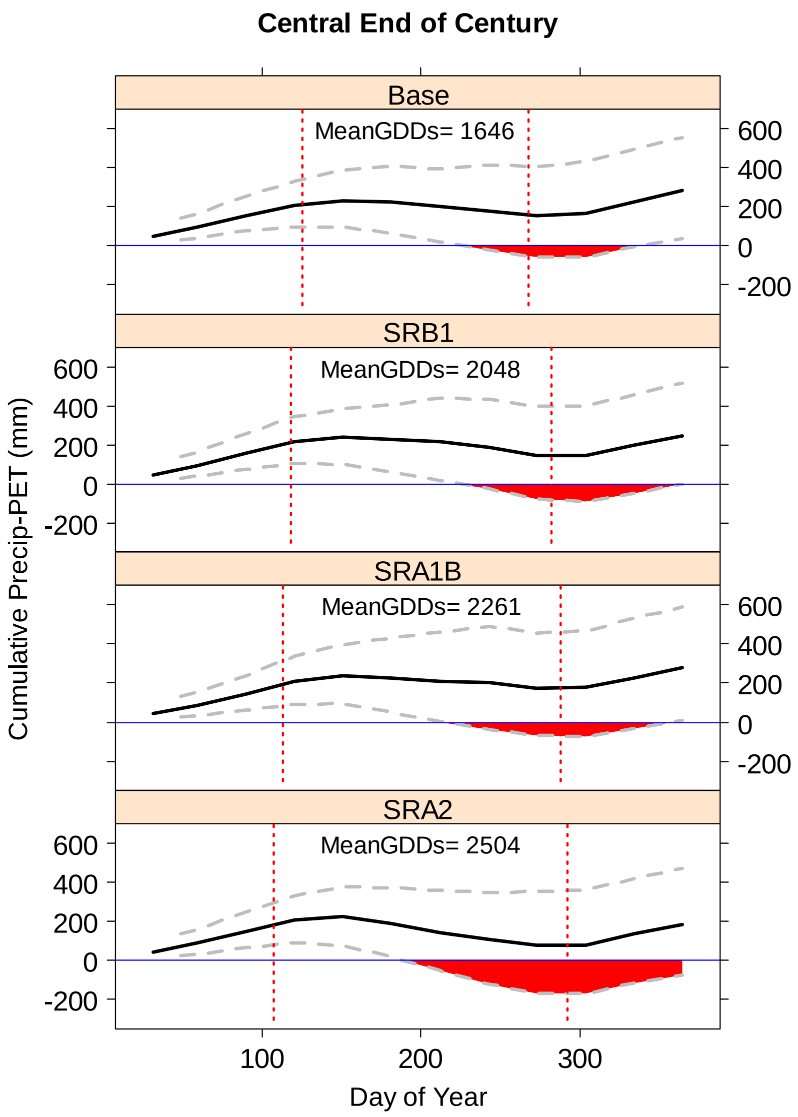
Central End of Century
Base
MeanGDDs= 1646
SRB1
MeanGDDs= 2048
SRA1B
MeanGDDs= 2261
SRA2
MeanGDDs= 2504
Cumulative Precip-PET (mm)
600
400
200
0
-200
100
200
300
Day of Year

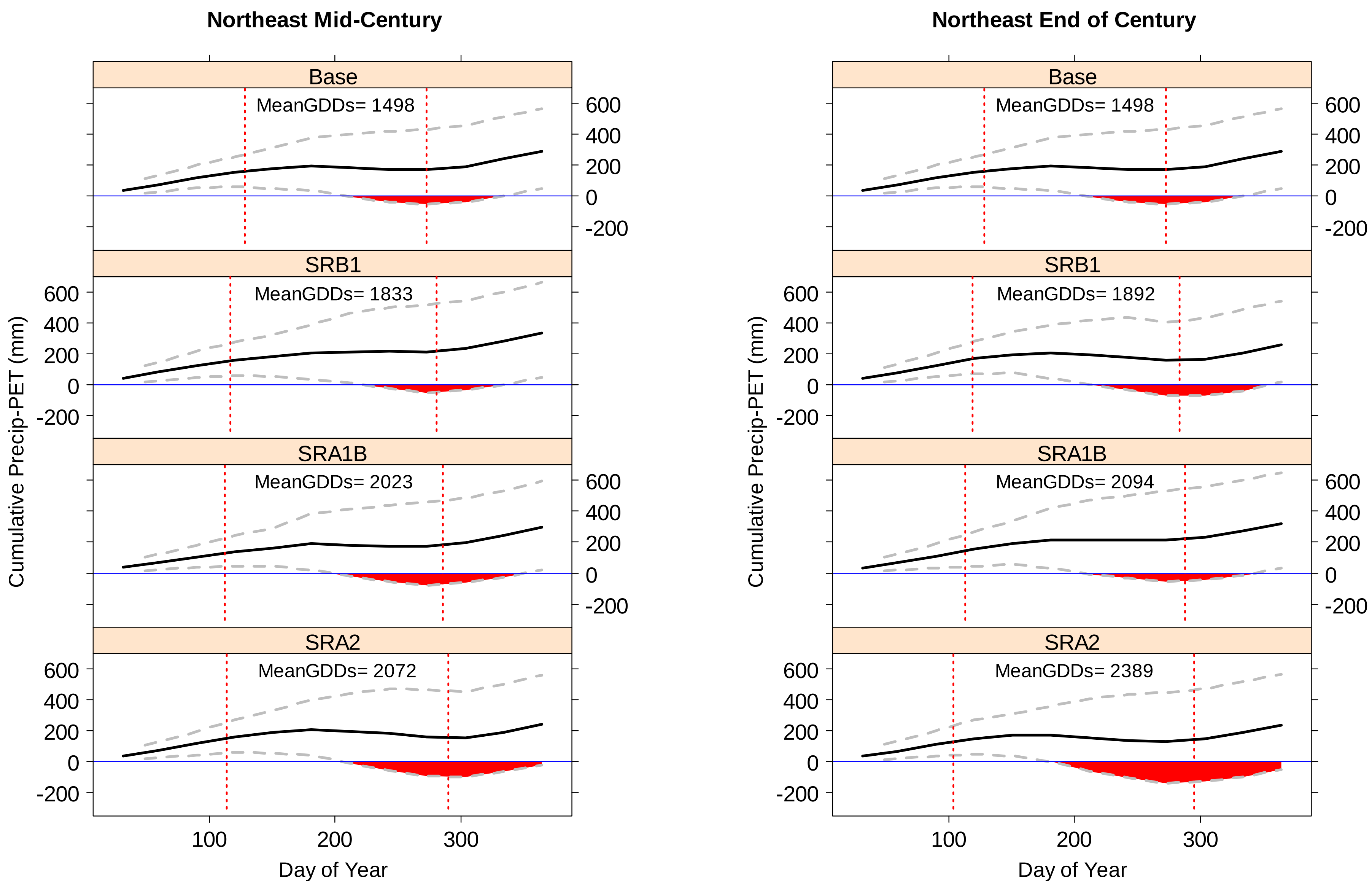

Northeast Mid-Century
Northeast End of Century
Base
SRB1
SRA1B
SRA2
MeanGDDs= 1498
MeanGDDs= 1833
MeanGDDs= 2023
MeanGDDs= 2072
MeanGDDs= 1498
MeanGDDs= 1892
MeanGDDs= 2094
MeanGDDs= 2389
Cumulative Precip-PET (mm)
Day of Year
600
400
200
0
-200
100
200
300

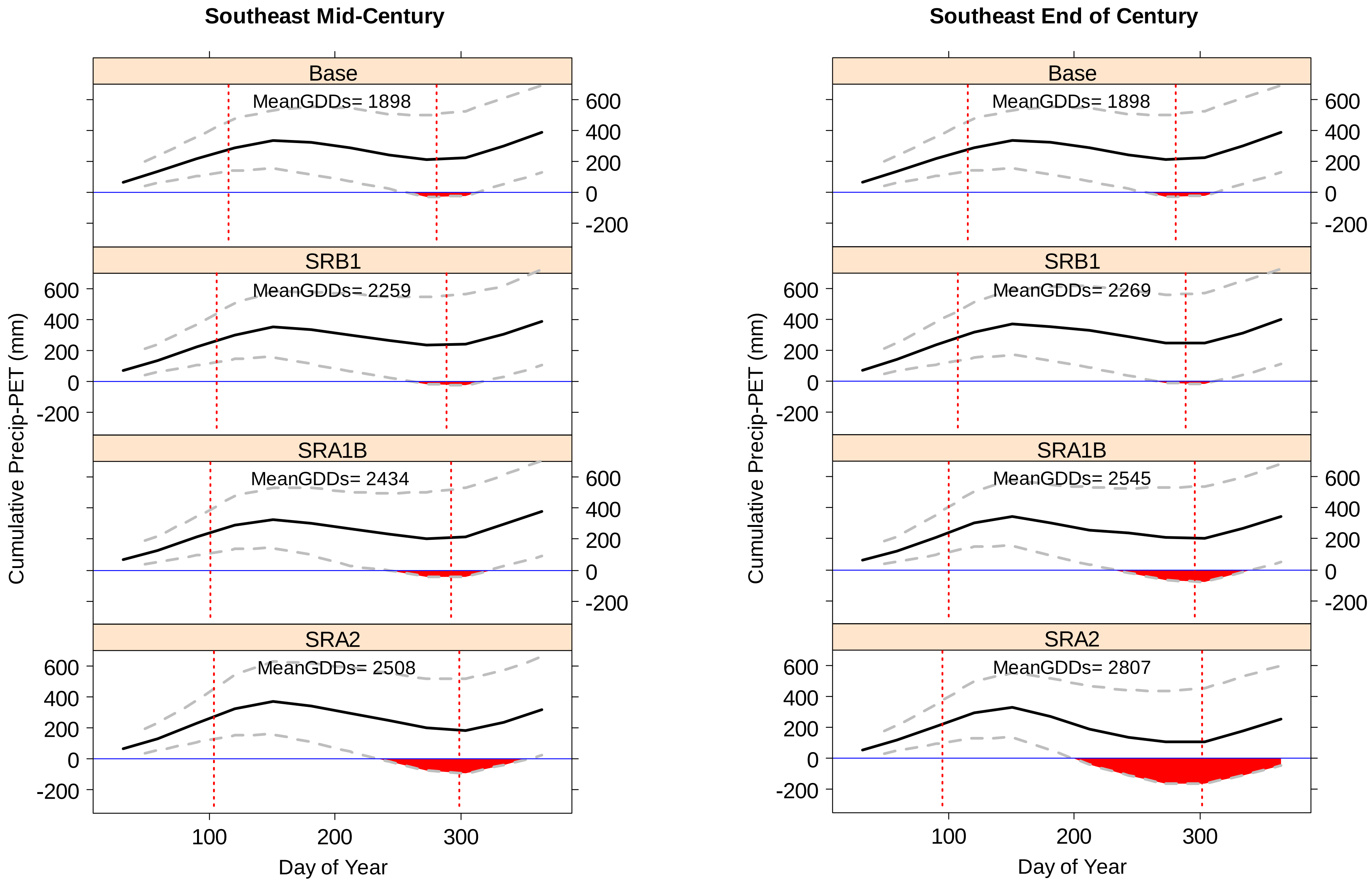

Southeast Mid-Century
Southeast End of Century
Base
MeanGDDs= 1898
SRB1
MeanGDDs= 2259
MeanGDDs= 2269
SRA1B
MeanGDDs= 2434
MeanGDDs= 2545
SRA2
MeanGDDs= 2508
MeanGDDs= 2807
600
400
200
0
-200
Cumulative Precip-PET (mm)
100
200
300
Day of Year

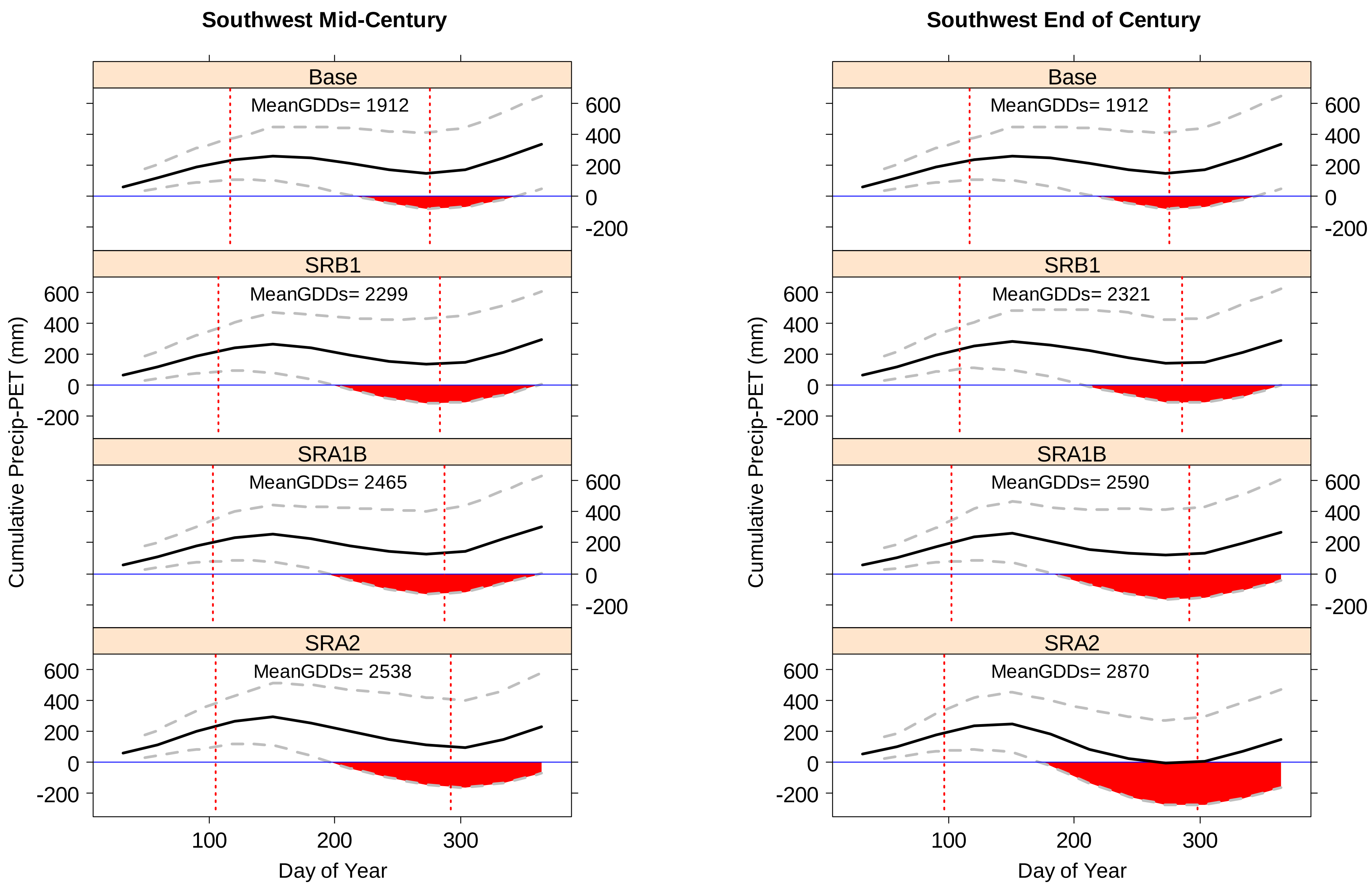

Southwest Mid-Century
Southwest End of Century
Base
MeanGDDs= 1912
SRB1
MeanGDDs= 2299
MeanGDDs= 2321
SRA1B
MeanGDDs= 2465
MeanGDDs= 2590
SRA2
MeanGDDs= 2538
MeanGDDs= 2870
Cumulative Precip-PET (mm)
Day of Year
600
400
200
0
-200
100
200
300

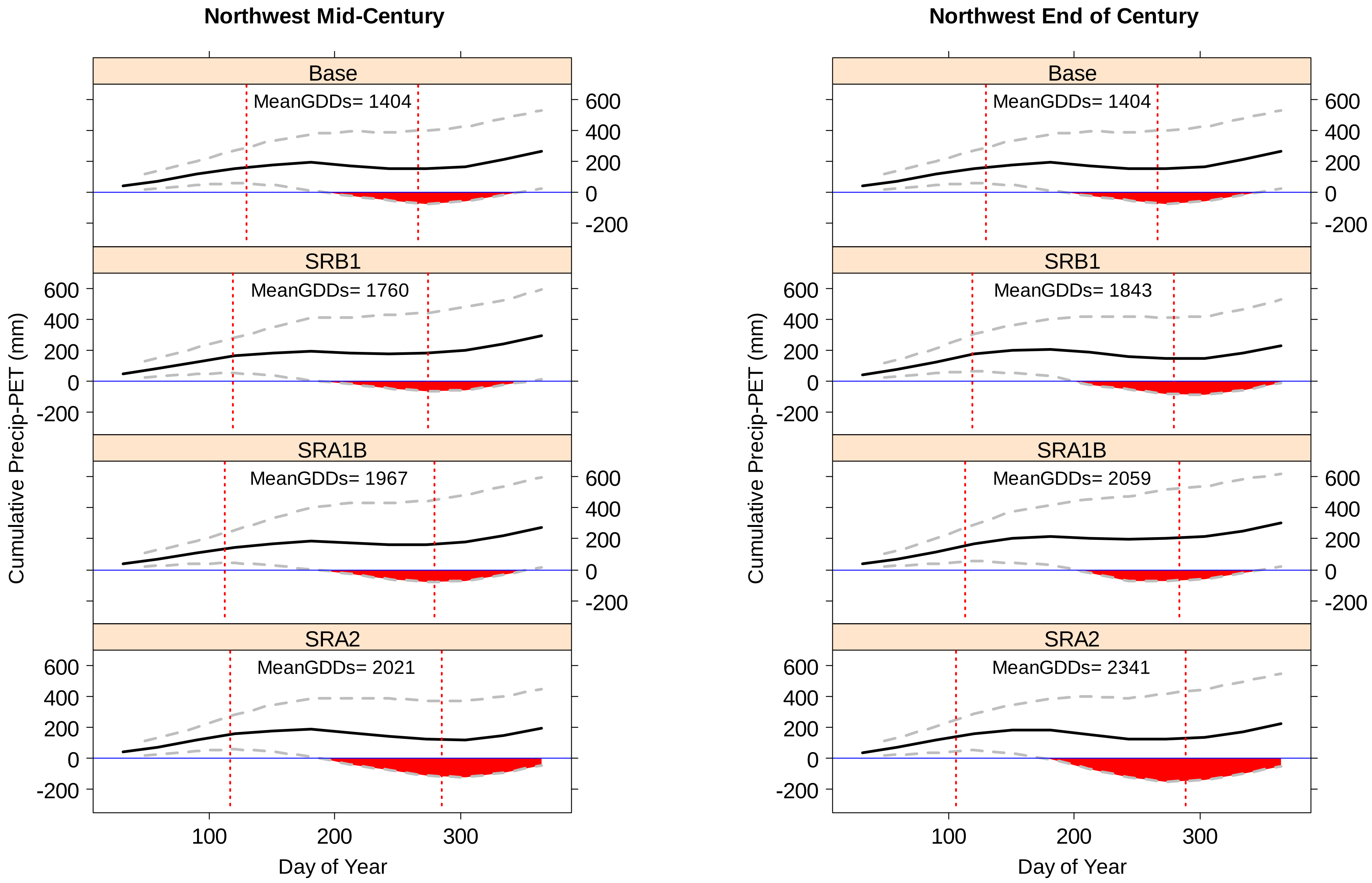
Northwest Mid-Century
Northwest End of Century
Base
SRB1
SRA1B
SRA2
MeanGDDs= 1404
MeanGDDs= 1760
MeanGDDs= 1967
MeanGDDs= 2021
MeanGDDs= 1404
MeanGDDs= 1843
MeanGDDs= 2059
MeanGDDs= 2341
Cumulative Precip-PET (mm)
600
400
200
0
-200
100
200
300
Day of Year

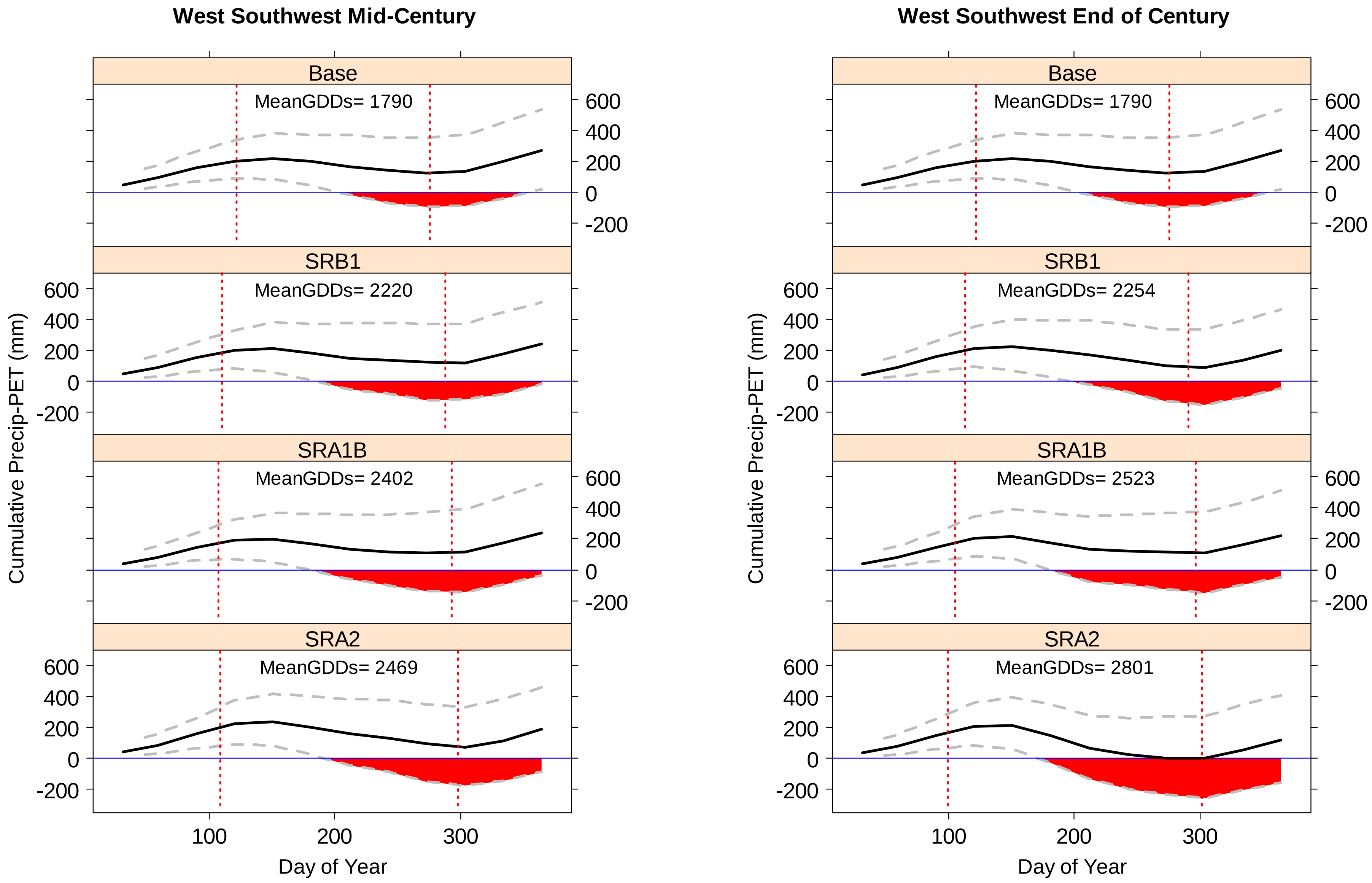
West Southwest Mid-Century
West Southwest End of Century
Base
SRB1
SRA1B
SRA2
MeanGDDs= 1790
MeanGDDs= 2220
MeanGDDs= 2402
MeanGDDs= 2469
MeanGDDs= 1790
MeanGDDs= 2254
MeanGDDs= 2523
MeanGDDs= 2801
Cumulative Precip-PET (mm)
600
400
200
0
-200
100
200
300
Day of Year

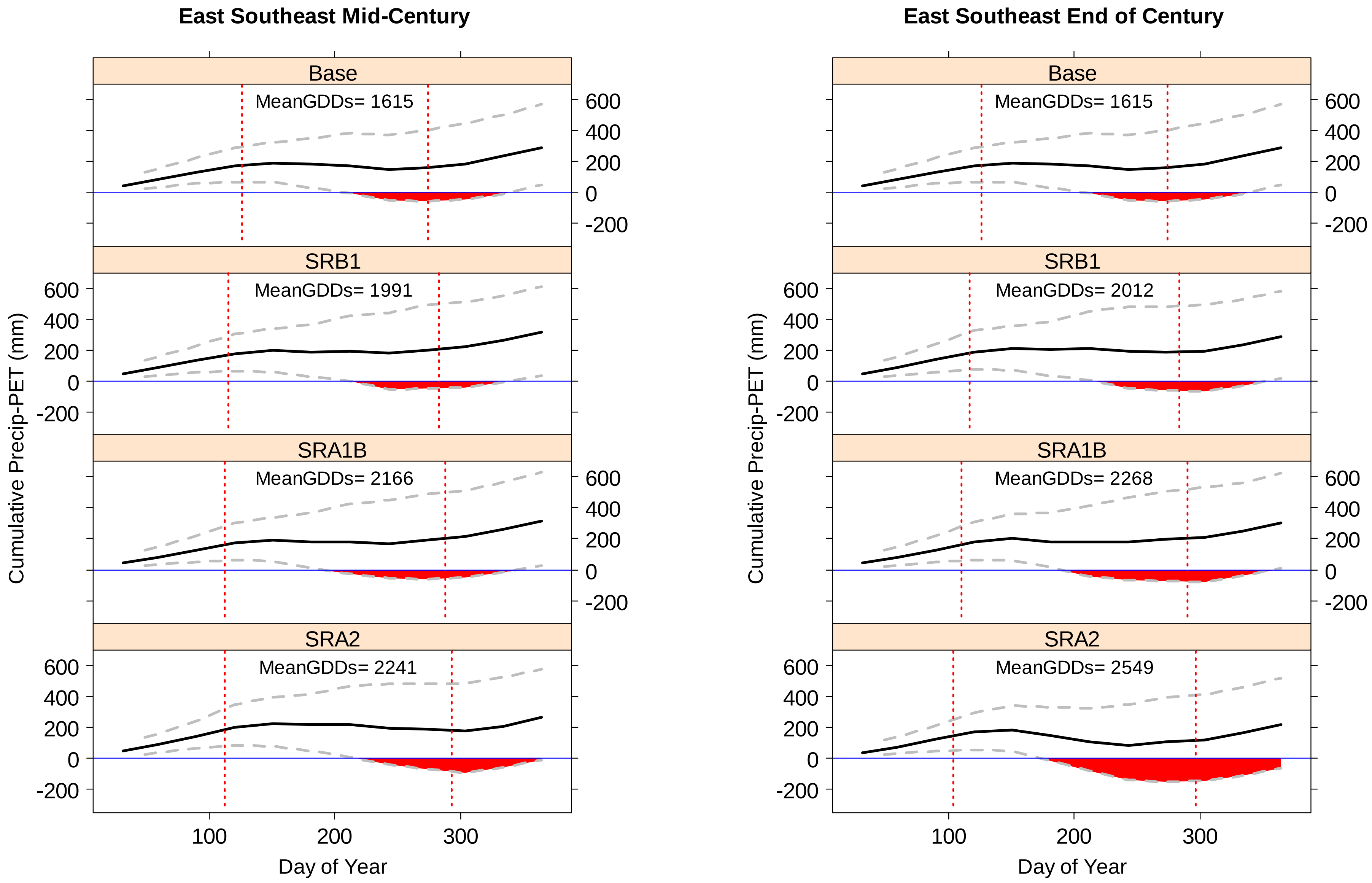
East Southeast Mid-Century
East Southeast End of Century
Base
MeanGDDs= 1615
SRB1
MeanGDDs= 1991
MeanGDDs= 2012
SRA1B
MeanGDDs= 2166
MeanGDDs= 2268
SRA2
MeanGDDs= 2241
MeanGDDs= 2549
Cumulative Precip-PET (mm)
600
400
200
0
-200
100
200
300
Day of Year

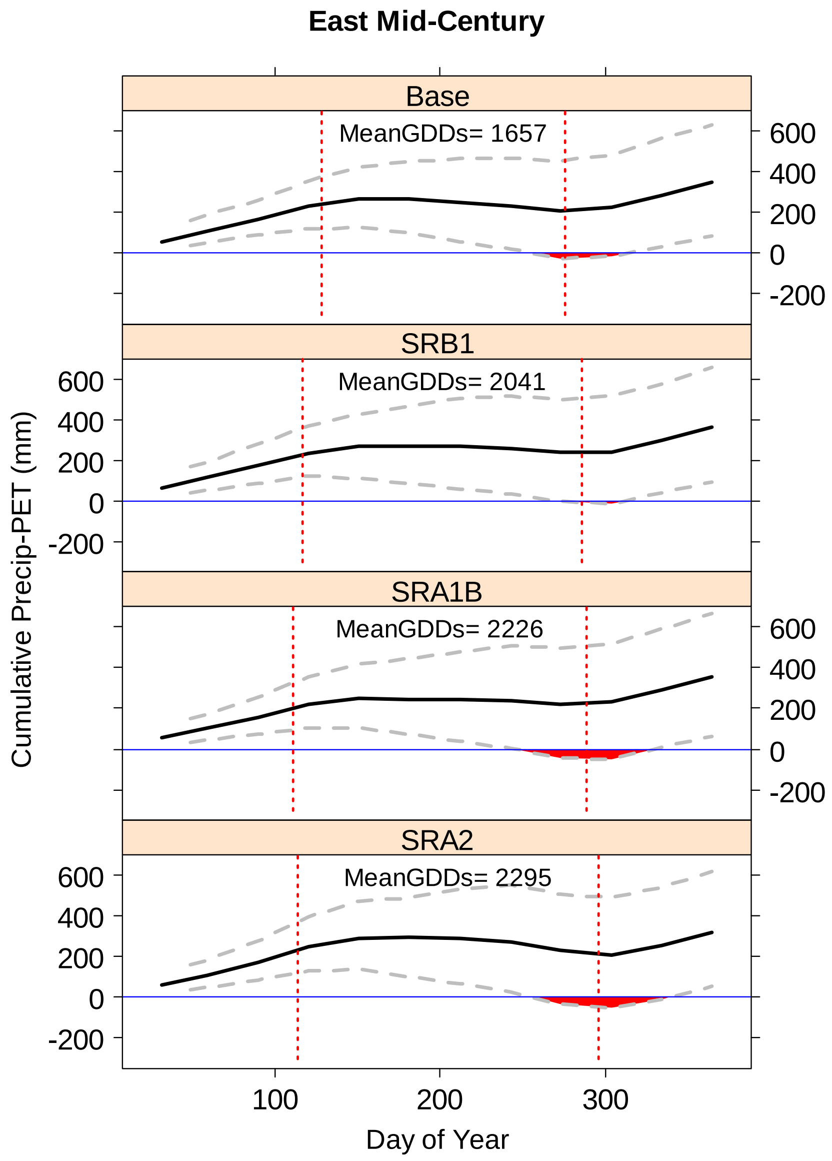
East Mid-Century
Base
MeanGDDs= 1657
SRB1
MeanGDDs= 2041
SRA1B
MeanGDDs= 2226
SRA2
MeanGDDs= 2295
Cumulative Precip-PET (mm)
Day of Year
600
400
200
0
-200
100
200
300

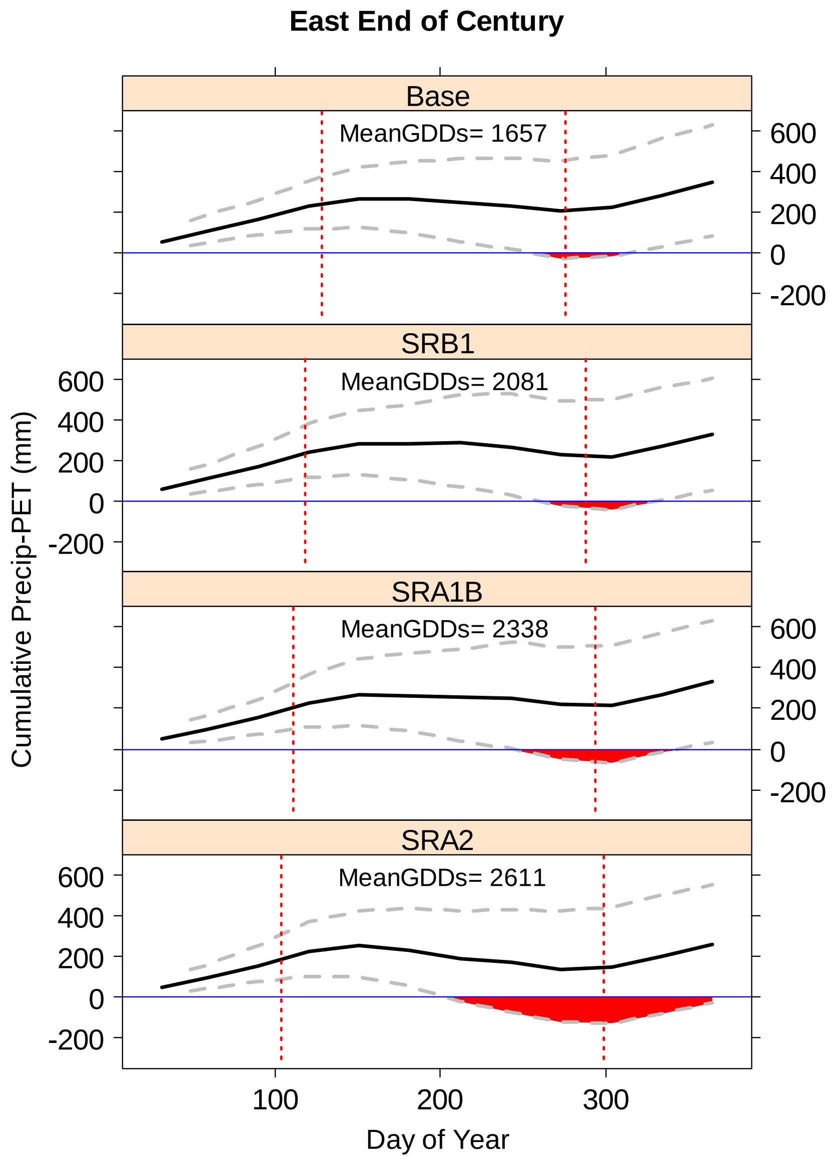
East End of Century
Base
MeanGDDs= 1657
SRB1
MeanGDDs= 2081
SRA1B
MeanGDDs= 2338
SRA2
MeanGDDs= 2611
Cumulative Precip-PET (mm)
Day of Year
600
400
200
0
-200
100
200
300

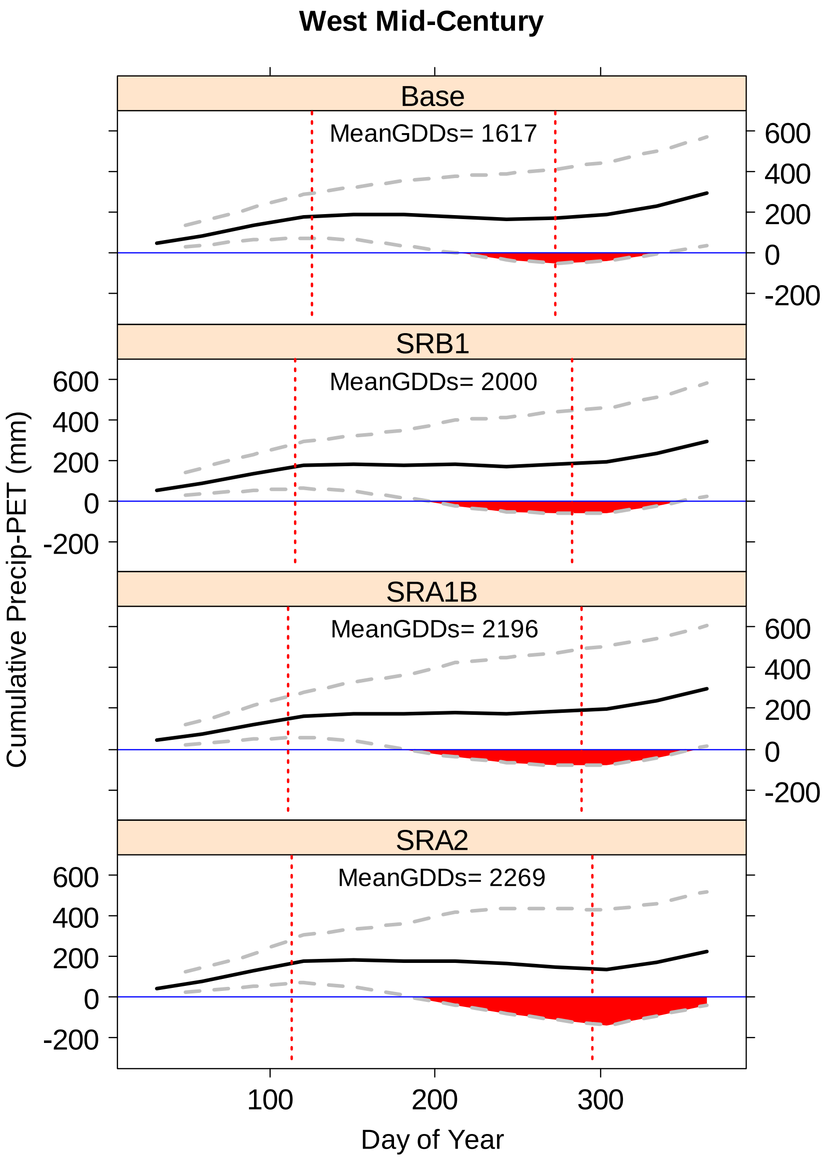
West Mid-Century
Base
MeanGDDs= 1617
SRB1
MeanGDDs= 2000
SRA1B
MeanGDDs= 2196
SRA2
MeanGDDs= 2269
600
400
200
0
-200
100
200
300
Cumulative Precip-PET (mm)
Day of Year

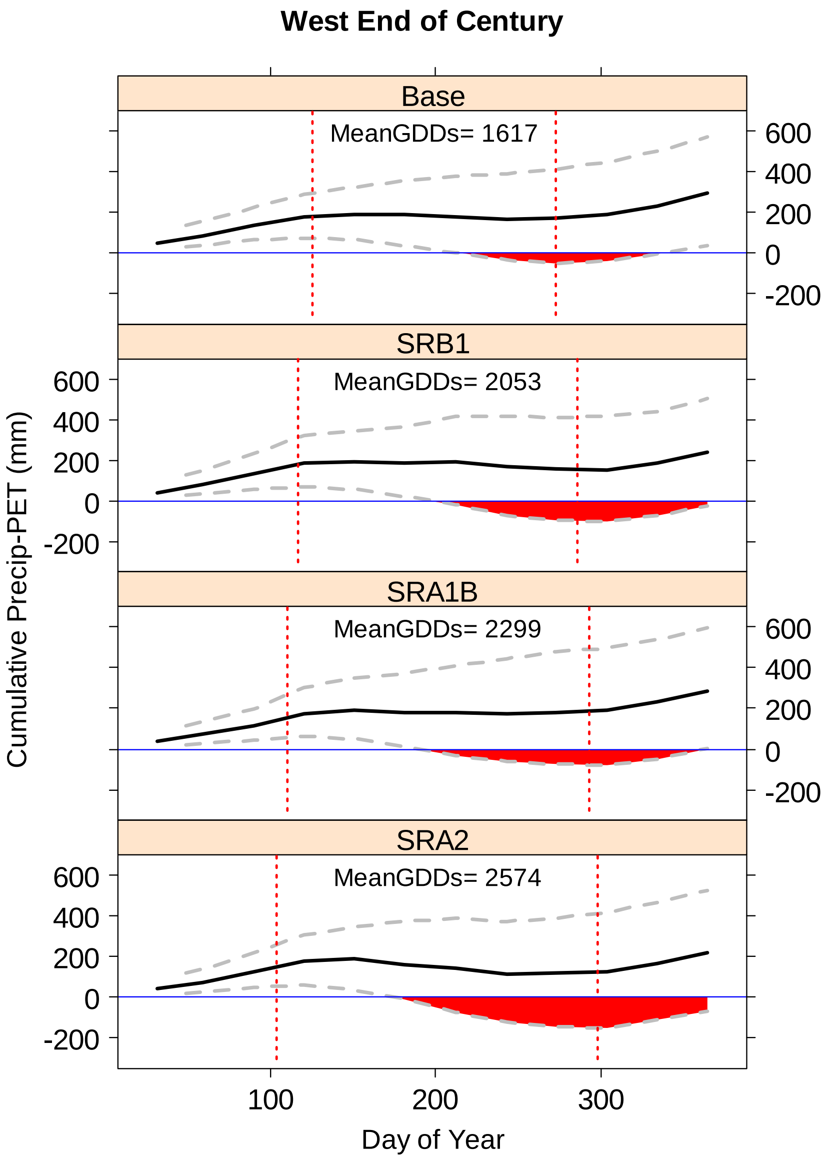
West End of Century
Base
MeanGDDs= 1617
SRB1
MeanGDDs= 2053
SRA1B
MeanGDDs= 2299
SRA2
MeanGDDs= 2574
600
400
200
0
-200
100
200
300
Cumulative Precip-PET (mm)
Day of Year
